# Long non-coding RNA *SNHG8* drives stress granule formation in tauopathies

**DOI:** 10.1038/s41380-023-02237-2

**Published:** 2023-09-21

**Authors:** Reshma Bhagat, Miguel A. Minaya, Arun Renganathan, Muneshwar Mehra, Jacob Marsh, Rita Martinez, Abdallah M. Eteleeb, Alissa L. Nana, Salvatore Spina, William W. Seeley, Lea T. Grinberg, Celeste M. Karch

**Affiliations:** 1 Department of Psychiatry, Washington University in St Louis, St Louis, MO USA; 2 Department of Neuroscience, Washington University in St Louis, St Louis, MO USA; 3 Department of Neurology, UCSF Weill Institute for Neurosciences, University of California, San Francisco, San Francisco, CA USA; 4 Department of Pathology, University of California, San Francisco, San Francisco, CA USA; 5 Department of Pathology, University of Sao Paulo, São Paulo, Brazil; 6 Knight Alzheimer Disease Research Center, Washington University in St Louis, St Louis, MO USA

**Keywords:** Neuroscience, Genetics, Stem cells, Psychiatric disorders, Molecular biology

## Abstract

Tauopathies are a heterogenous group of neurodegenerative disorders characterized by tau aggregation in the brain. In a subset of tauopathies, rare mutations in the *MAPT* gene, which encodes the tau protein, are sufficient to cause disease; however, the events downstream of *MAPT* mutations are poorly understood. Here, we investigate the role of long non-coding RNAs (lncRNAs), transcripts >200 nucleotides with low/no coding potential that regulate transcription and translation, and their role in tauopathy. Using stem cell derived neurons from patients carrying a *MAPT* p.P301L, IVS10 + 16, or p.R406W mutation and CRISPR-corrected isogenic controls, we identified transcriptomic changes that occur as a function of the *MAPT* mutant allele. We identified 15 lncRNAs that were commonly differentially expressed across the three *MAPT* mutations. The commonly differentially expressed lncRNAs interact with RNA-binding proteins that regulate stress granule formation. Among these lncRNAs, *SNHG8* was significantly reduced in a mouse model of tauopathy and in FTLD-tau, progressive supranuclear palsy, and Alzheimer’s disease brains. We show that *SNHG8* interacts with tau and stress granule-associated RNA-binding protein TIA1. Overexpression of mutant tau in vitro is sufficient to reduce *SNHG8* expression and induce stress granule formation. Rescuing *SNHG8* expression leads to reduced stress granule formation and reduced TIA1 levels in immortalized cells and in *MAPT* mutant neurons, suggesting that dysregulation of this non-coding RNA is a causal factor driving stress granule formation via TIA1 in tauopathies.

## Introduction

Tauopathies are a class of neurodegenerative diseases that manifest as cognitive decline and are neuropathologically characterized by the accumulation of intracellular hyperphosphorylated tau protein [1]. Dominantly inherited mutations in the *MAPT* gene, which encodes the tau protein, are sufficient to cause disease in a subset of tauopathies termed frontotemporal lobar degeneration with tau pathology (FTLD-tau) [2]. However, the underlying mechanisms by which *MAPT* mutations cause disease remain unclear.

Several mechanisms contributing to FTLD-tau have been proposed. *MAPT* mutations have been reported to affect molecular and structural properties of tau. As a consequence, microtubule binding efficiency, post-translational modification status, and isoform balance of tau in the central nervous system (CNS) may be altered [3]. *MAPT* mutations also lead to tau accumulation, impaired neuronal function, cell death, mitochondrial stress, autophagic and lysosomal dysregulation, and nuclear-cytosolic transport defects [4–9]. Whether there are mechanisms upstream of these molecular events remains poorly understood.

Disruption of non-coding regulatory elements in the genome may have broad downstream effects that have yet to be fully explored in FTLD-tau [10, 11]. Stem cell modeling along with genome editing have revealed that *MAPT* mutations are sufficient to elicit a number of molecular events associated with synaptic function and proteostasis [5, 12–15]. These studies have focused on understanding the effects of *MAPT* mutations on coding genes. Yet, coding genes represent only 2% of the human genome. Non-coding regions, such as long non-coding RNAs (lncRNAs), represent 31.79% of the genome. LncRNAs play crucial regulatory roles in many cellular processes [16], including the regulation of transcriptional modulation, post-transcriptional control, nuclear-cytoplasmic transport, translational inhibition, mRNA stability, RNA decoys, and regulation of protein activity [17]. LncRNAs also interact with a wide range of RNA-binding proteins, including those involved in stress granule formation [18, 19]. With non-coding RNAs making up a significant portion of the human genome, the impact of *MAPT* mutations on lncRNAs is an unexplored area that may hold key insights into the underlying mechanisms of FTLD-tau.

Our findings suggest that *MAPT* mutations have a significant impact on lncRNA expression in human neurons. We identified a lncRNA, *SNHG8*, that is reduced across three types of *MAPT* mutations and reduced in brains from tauopathy mouse models and human patients. In vitro studies demonstrate that *MAPT* mutations disrupt *SNHG8* expression, which promotes stress granule formation. This represents a novel mechanism that could be targeted for therapeutic intervention in the context of tauopathies. These results highlight the importance of studying the role of lncRNAs in the regulation of stress granule formation and the effects of *MAPT* mutations on lncRNA expression in the development of effective treatments for tauopathies.

## Material and methods

### Patient consent

To obtain fibroblasts, skin punches were performed following written informed consent from the donor. The informed consent was approved by the Washington University School of Medicine Institutional Review Board and Ethics Committee (IRB 201104178 and 201306108).

The University of California San Francisco Institutional Review Board approved the operating protocols of the UCSF Neurodegenerative Disease Brain Bank (from which brain tissues were obtained). Participants or their surrogates provided consent for autopsy, in keeping with the guidelines put forth in the Declaration of Helsinki, by signing the hospital’s autopsy form. If the participant had not provided future consent before death, the DPOA or next of kin provided it after death. All data were analyzed anonymously.

### iPSC generation and genome engineering

Human iPSCs used in this study have been previously described [20]. iPSC lines were generated using non-integrating Sendai virus carrying the Yamanaka factors: OCT3/4, SOX2, KLF4, and cMYC (Life Technologies) [21, 22]. The following parameters were used for the characterization of each of the iPSC lines using standard methods [21]: pluripotency markers by immunocytochemistry (ICC) and quantitative PCR (qPCR); spontaneous or TriDiff differentiation into the three germ layers by ICC and qPCR; assessment of chromosomal abnormalities by karyotyping; and *MAPT* mutation status confirmation by Sanger sequencing (characterization data previously reported [15]).

To determine the impact of the *MAPT* mutant allele on molecular phenotypes, we used CRISPR/Cas9-edited isogenic controls in which the mutant allele was reverted to the wild-type (WT) allele in each of the donor iPSC lines as previously described [15, 20]. The resulting edited iPSC lines were characterized as described above in addition to on- and off-target sequencing (characterization data previously reported [15]). All iPSC lines used in this study carry the *MAPT* H1/H1 common haplotype. All cell lines were confirmed to be free of mycoplasma.

### Differentiation of iPSCs into cortical neurons

iPSCs were differentiated into cortical neurons as previously described [5, 20] (10.17504/protocols.io.p9kdr4w). Briefly, iPSCs were plated at a density of 65,000 cells per well in neural induction media (StemCell Technologies) in a 96-well v-bottom plate to form neural aggregates. After 5 days, cells were transferred into culture plates. The resulting neural rosettes were isolated by enzymatic selection (Neural Rosette Selection Reagent; StemCell Technologies) and cultured as neural progenitor cells (NPCs). NPCs were differentiated in planar culture in neuronal maturation medium (neurobasal medium supplemented with B27, GDNF, BDNF, and cAMP). The cells were analyzed after 6 weeks in neuronal maturation medium. At this time, tau protein levels are stable and similar to protein profiles described in human brains [23].

### RNA sequencing and lncRNA transcript quantification

RNAseq was generated from iPSC-derived neurons as previously described [12, 15]. Briefly, samples were sequenced by an Illumina HiSeq 4000 Systems Technology with a read length of 1 × 150 bp and an average library size of 36.5 ± 12.2 million reads per sample.

Salmon (v. 0.11.3) [24] was used to quantify the expression of the genes annotated within the human reference genome (GRCh38.p13; Supplementary Table 1). The lncRNA genes were selected for downstream analyses. LncRNA genes that were present in at least 10% of samples with expression >0.1 TPM were included in subsequent analyses: 7,537 lncRNA genes (Supplementary Fig. 1).

### Principal component and differential expression analyses

Principal component analyses (PCA) were performed with the selected 7,537 non-coding genes using regularized-logarithm transformation (rlog) counts. Differential gene expression was performed using the DESeq2 (v.1.22.2) R package [25]. PCA and differential gene expression analyses were performed independently for each pair of *MAPT* mutations and isogenic controls. Each *MAPT* mutation and its isogenic control were considered independent cohorts due to their shared genetic background. PCA and Volcano plots were created for each comparison using the ggplot2 R package (v3.3.6) [26].

### Functional annotation of differentially expressed lncRNA genes

LncSEA was used to determine the RNA-binding protein interactions of common differentially expressed lncRNAs [27]. Gene relationships of top RNA-binding proteins and *MAPT*, including physical interaction, co-localization, pathway, shared protein domain, and genetic interaction, were examined using the GeneMANIA Cytoscape plugin [28].

CatRAPID was applied to identify the interactions between individual lncRNAs and RNA-binding proteins [29, 30]. The input for CatRAPID analysis was the FASTA sequence of lncRNA and protein. The output was a heat map where the axes represent the indexes of the RNA and protein sequences with interaction propensity and discriminative power. The Interaction Propensity is a measure of the interaction probability between one protein (or region) and one RNA (or region). This measure is based on the observed tendency of the components of ribonucleoprotein complexes to exhibit specific properties of their physio-chemical profiles that can be used to make a prediction. The Discriminative Power is a statistical measure introduced to evaluate the Interaction Propensity with respect to CatRAPID training. It represents confidence of the prediction. The Discriminative Power (DP) ranges from 0% (unpredictable) to 100% (predictable). DP values above 50% indicate that the interaction is likely to take place, whereas DPs above 75% represent high-confidence predictions.

### Plasmids

Plasmids pRK5-EGFP containing 4R0N Tau WT or P301L (Addgene plasmids 46904 and 46908) were used to evaluate the impact of tau on stress granule formation and lncRNA expression [31]. To test the impact of *SNHG8* on stress granule formation, a plasmid containing human *SNHG8* (transcript 203) in pcDNA3.1(+)-C-eGFP was used (pcDNA3.1(+)-SNHG8-203-EGFP (transcript 203) and control pcDNA3.1(+)-EGFP; Genescript). Untagged P301L-Tau (4R2N) constructs in pcDNA3.1(+) were employed in tau interaction and SNHG8-EGFP rescue experiments [32].

### Transient transfection in HEK293-T cells

HEK293-T cells were grown in Dulbecco’s Modified Eagle Medium (DMEM; Life Technologies) supplemented with 10% FBS, 1% L-Glutamine, and 1% Penicillin and streptomycin solution. Plasmids were transfected using Lipofectamine 2000 (Invitrogen, San Diego, CA, USA) according to the manufacturer’s protocol. The transfected cells were evaluated after 24 or 48 h for immunocytochemistry or RNA-immunoprecipitation, respectively.

### RNA immunoprecipitation

RNA-immunoprecipitation (RIP) was performed as previously described with minor modifications [33]. Briefly, HEK293-T cells transfected with either WT-Tau (2N4R) or control vector plasmid constructs [32]. Cells were lysed in RIP buffer containing 20 mM Tris-HCl, pH 8.0, 200 mM NaCl, 1 mM EDTA, 1 mM EGTA, 0.5% Triton X-100, 0.4U/ul RNase inhibitor and 1X protease inhibitor cocktail (Roche). The lysate was pre-cleared by centrifugation and the total protein of the supernatant was quantified by BCA protein assay kit (Pierce). Approximately 1 mg of pre-cleared lysate was incubated with 2.5 ug of Tau5 and Tau7 antibodies (generous gift from Lester Binder) or pre-immune IgG (sc-2025) overnight at 4 °C. The lncRNA-protein complexes were captured with antibody coupled protein A/G beads (Thermo Fisher Scientific, Cat#20333), washed with RIP buffer, and treated with RNase free DNase I. RNAs were isolated using the Trizol method. QPCR was performed with *SNHG8* and *GAPDH* primer by using iTaq-one step RT-PCR kit (Bio-Rad).

For western blot analyses, approximately 20% of the capture beads were washed three times with cell lysis buffer and once with 1X phosphate-buffered saline (PBS). The washed beads were mixed with 4x Laemmli sample buffer (Bio-Rad, Cat#: 161-0747) and 10% β-mercaptoethanol, heated at 95 °C for 30 min, and run on a 4%–12% Bis-Tris gel (NuPAGE). Proteins were transferred to PVDF membrane and blocked for 1 h at room temperature in 5% milk in phosphate buffered saline with 0.1% Tween 20 (PBS-T). Membranes were probed with the mouse anti-Tau5 antibody (1:2000; Abcam, Cat# ab3931, RRID: AB_304171) and GAPDH (1:5000, Thermo Fisher Scientific, Cat# MA5-15738, RRID: AB_10977387) overnight at 4 °C. Membranes were subsequently washed and incubated in affiniPure Goat anti-mouse HRP (1:3000, Jackson Immuno Research Labs, Cat# 115-035-174, RRID: AB_2338512) for 1 h at room temperature, washed, and developed using SuperSignal West Pico PLUS Chemiluminescent Substrate (Thermo Fisher Scientific).

### Mouse model of tauopathy

To evaluate whether the genes differentially expressed in iPSC-derived neurons from *MAPT* mutation carriers were altered in animal models of tauopathy, we analyzed transcriptomic data from a Tau-P301L mouse model of tauopathy and non-transgenic controls [34, 35]. Differential gene expression of lncRNAs was performed in mice at 2, 4, and 8 months of age using unpaired t-tests to assess significance.

### Gene expression analysis in PSP and AD brains

To determine whether the differentially expressed lncRNAs in the *MAPT* mutant iPSC-derived neurons capture molecular processes that occur in human brains with primary tauopathy, we analyzed gene expression in a publicly available dataset: the temporal cortex of 76 control, 82 PSP, and 84 AD brains (syn6090813) [36]. Differential gene expression analyses comparing controls with PSP and AD brains were performed using a “Simple Model” that employs multi-variable linear regression analyses using normalized gene expression measures and corrected by sex, age-at-death, RNA integrity number (RIN), brain tissue source, and flowcell as covariates [36]. Transcriptomic data from the middle temporal gyrus of FTLD-tau patients with *MAPT* IVS10 + 16 and p.P301L mutation (*MAPT* IVS10 + 16 *n* = 2 and *MAPT* p.P301L *n* = 1) and neuropathology free controls (*n* = 3) were also analyzed [15]. Differential expression analyses comparing FTLD-tau mutation carrier brains with controls were performed using DESeq2 (v.1.22.2) R package [25] as previously described [15].

### qPCR validation of lncRNA-SNHG8 and TIA1

*SNHG8* expression was validated using qPCR by SYBR green chemistry. Specific primers probes (Supplementary Table 2) were used to study the expression of lncRNAs in neurons expressing the *MAPT* IVS10 + 16, p.R406W, or p.P301L mutation along with their isogenic controls. Transcript quantification of *TIA1* from HEK293-T cells under stress or basal conditions was performed using specific primers to *TIA1* (Supplementary Table 2). LncRNA expression was measured by qPCR on a Quantstudio 3 qPCR machine (Applied Biosystems by Thermo Fisher Scientific) using specific primers. Melt curve was analyzed to study the specificity of the primers.

### Induction and quantification of stress granules

TIA1, G3BP2, or PABP were used to monitor stress granule formation [37]. Stress granule formation was induced by culturing HEK293-T cells in a nutrient poor buffer (Hank’s buffer) or 0.5 mM sodium arsenite, which induces oxidative stress, as previously described [38–41]. HEK293-T or iPSC-derived neurons were immunostained with TIA1 (Sigma Aldrich-SAB4301803, 1:250 dilution), G3BP2 (Cell Signaling Technology- 31799S, 1:500 dilution), or PABP (Sant Cruz-sc-32318, 1:50 dilution) antibodies. Briefly, to perform immunocytochemistry, cells were grown on chamber slides. Culture media was aspirated, and cells were washed with PBS and fixed with 4% paraformaldehyde (Sigma) for 20 min at room temperature. Cells were washed with PBS and incubated with permeabilization buffer (0.1% Triton X-100 in PBS). Cells were then blocked in 0.1% bovine serum albumin (BSA; Sigma) and treated with primary and secondary antibodies diluted in 0.1% BSA. Immunostained cells were imaged (BZ-X800 series, Keyence fluorescent microscope, Keyence, IL, USA and Zeiss LSM 980 with Airyscan 2, Zeiss, Germany). At least six random images were captured per replicate, per condition. To calculate the percentage of cells positive for stress granules, the number of cells with stress granules in GFP-positive cells were divided by total number of GFP-positive cells. To determine the number of stress granules/cell and total stress granules in all GFP-positive cells, TIA1-, G3BP2-, and PABP-positive inclusions were manually counted and corrected for the GFP-positive cells.

### RNAscope

RNAscope (Advanced Cell Diagnostics, ACD; Hayward, CA) was performed using BaseScope Reagent Kit v2 – RED (323900) kit by using specific probes targeting human *SNHG8* (NC_000004.12) according to the manufacturer’s protocol. 3ZZ probe named BA-Hs-SNHG8-O1-3zz-st targeting 2-133 of NC_000004.12:118278708-118279137 was used. BaseScope is a chromogenic assay: red chromogen was used for *SNHG8* detection which can be seen under a fluorescent microscope in the Texas Red spectrum. HEK293-T cells were fixed with paraformaldehyde and washed with PBS. Slides were then hybridized with target probes and incubated in a HybEZ oven (ACD) for 2 h at 40 °C. Next, signals were amplified and generated with a BaseScope Detection Reagent Kit v2 – RED. Cells were then counterstained with DAPI. *SNHG8* expression was scored as positive if staining was present in HEK293-T cells. For visualizing the slides stained with *SNHG8*, a Keyence microscope (BZ-X800 series, Keyence fluorescent microscope, Keyence, IL, USA) and confocal microscope (Zeiss LSM 980 with Airyscan 2, Zeiss, Germany) were used. Images were captured at 40X and 60X magnification. ImageJ (https://imagej.nih.gov/) was used to quantify the mean intensity of *SNHG8*. The freehand selections tool was used to mark the transfected cells in green channel and the measure tool was used to quantify the *SNHG8* signal in the red channel.

### Overexpression of SNHG8 in iPSC-derived neurons

NPCs expressing the *MAPT* p.P301L mutation were nucleofected with GFP vector or *SNHG8*-GFP containing vector using the manufacturer’s instructions. Briefly 3ug of plasmid was nucleofected in 1 × 10^6^ NPCs using the Lonza DC-104 program and cells were plated onto PLO/Lamin-coated plate in Neural Induction Media (StemCell Technologies) with 10% FBS. After cells recovered from nucleofection, cells were differentiated into neurons as described above. At day 20 of neural differentiation, cells were plated on the coated 8 well chamber slides at density of 50,000/well. At day 42, cells were fixed and processed for immunocytochemistry.

### Statistical analysis

Statistical analyses of biochemical and immunocytochemistry experiments were performed using GraphPad Prism version 9.2.0 (332) software. Each experiment was performed at least three times to determine statistical significance. Data distribution was assumed to be normal. Comparison between experimental and control group was analyzed using Student’s *t* test, a level of *p* < 0.05 was considered statistically significant. Details of the sample sizes and statistical tests used are indicated in the figure legends.

## Results

### *MAPT* mutations are sufficient to drive changes in lncRNAs in human neurons

**Fig. 1:**
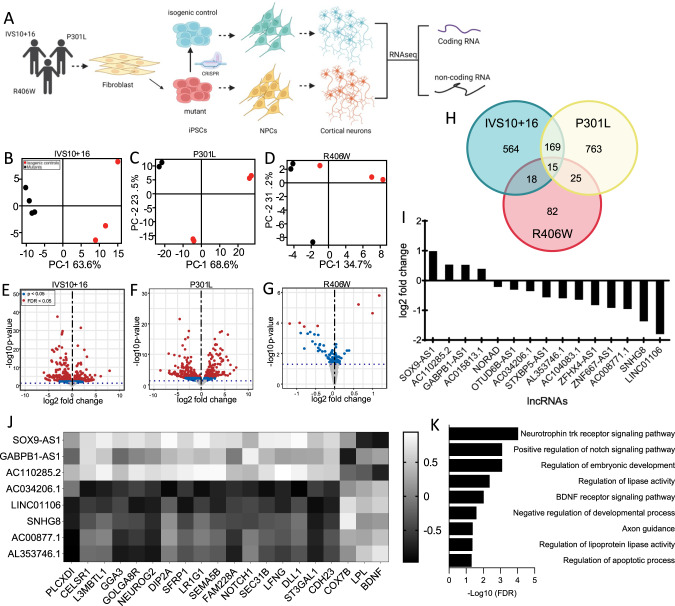
Mutations in *MAPT* are sufficient to drive changes in long non-coding RNA (lncRNA) profiles in iPSC-derived neurons. **A** Diagram of experimental design. **B**–**D** Principal component analysis (PCA) of *MAPT* IVS10 + 16, p.P301L, and p.R406W carriers and their respective isogenic controls using only lncRNAs. Red dots, CRISPR-corrected isogenic controls. Black dots, *MAPT* mutation carriers. **E**–**G** Volcano plots representing the differential expression of lncRNAs in *MAPT* IVS10 + 16, p.P301L, and p.R406W carriers compared to their respective isogenic controls. Red dots, differentially expressed genes (FDR < 0.05). Blue dots, differentially expressed genes (*p* < 0.05). Gray dots, not significant. **H** Venn diagram showing lncRNA overlap among all three *MAPT* mutations. **I** Bar graph representing mean log2 foldchange of common differentially expressed lncRNAs. **J** Heat map of correlation between differentially expressed lncRNAs and differentially expressed protein coding RNA. Correlation coefficient >0.6. **K** GO terms from the analysis of highly correlated protein coding RNAs.

To explore the contribution of lncRNAs to tauopathy, we examined whether there were a common set of lncRNAs that are downstream of *MAPT* mutations. The more than 50 *MAPT* mutations fall into three major classes: (1) intronic mutations that alter splicing, leading to an imbalance in tau isoforms; (2) missense mutations within exon 10, leading to mutations in only a subset of tau isoforms; (3) missense mutations occurring in all tau isoforms. To begin to define the common non-coding mechanisms driving FTLD-tau, we have studied *MAPT* mutations that fall into each of the three major classes: *MAPT* IVS10 + 16, p.P301L, and p.R406W, respectively. Transcriptomic data from iPSC-derived neurons carrying *MAPT* IVS10 + 16, p.P301L, or p.R406W together with their CRISPR/Cas9-generated isogenic controls were analyzed (Fig. 1A). We have previously demonstrated that these *MAPT* mutant neurons produce elevated phosphorylated tau, endolysosomal defects, and molecular signatures consistent with those identified in human FTLD-tau brains [12, 15, 20, 42, 43].

To define the global impact of *MAPT* mutations on lncRNAs across the genome, we performed differential expression analyses. Principal component analysis revealed that variation in the lncRNA transcriptome was sufficient to distinguish *MAPT* mutations from their isogenic controls (Fig. 1B–D). Differential expression analyses identified a number of lncRNAs changing as a function of the presence of the mutant allele (Fig. 1E–G; Supplementary Table 3; *p* < 0.05; *MAPT* IVS10 + 16 (*n* = 766; Supplementary Table 4), p.P301L (*n* = 972; Supplementary Table 5), and p.R406W (*n* = 141; Supplementary Table 6)). Among these, 15 lncRNAs were significantly altered in the same direction across the three datasets (Fig. 1H–I; Supplementary Table 7). Thus, *MAPT* IVS10 + 16, p.P301L, and p.R406W mutations were sufficient to shift the lncRNA transcriptomic state of human neurons, and three classes of *MAPT* mutations generated a common molecular signature that we sought to further explore for their role in pathologic processes.

### Mutant tau-regulated lncRNAs disrupt the expression of coding genes in human neurons

To begin to define the regulatory role of the 15 common lncRNAs, we evaluated the impact of these lncRNAs on coding gene expression. We have previously reported that these three classes of *MAPT* mutations are sufficient to drive altered gene expression of 275 protein coding genes (*p* < 0.05; [15]. LncRNAs can act in a cis or trans manner to inhibit or activate transcription of protein coding genes [44]. We defined the coding genes that are proximal (<5 kb) to the lncRNAs (Supplementary Table 8). We also asked whether expression of the 15 common lncRNAs were correlated with expression of the 275 protein coding genes (Fig. 1J). We found that 8 of the 15 lncRNAs were highly correlated with 20 of the 275 protein coding genes: *SOX9-AS1*, *GABPB1-AS1*, *AC110285.2*, *AC034206.1*, *LINC01106*, *SNHG8*, *AC00877.1*, *AL353746.1* (Fig. 1J; Supplementary Table 9). Gene enrichment analyses were then performed to determine the biological role of these regulatory relationships. The protein coding genes highly correlated with the differentially expressed lncRNAs were found to be enriched in pathways related to Neurotrophin trk receptor signaling (FDR = 9.22 × 10^−5^), Notch signaling (FDR = 4.8 × 10^−2^), BDNF signaling (FDR = 9.5 × 10^−3^), lipoprotein lipase activity (FDR = 4.3 × 10^−3^), and axonal guidance (FDR = 4.17 × 10^−2^) (Fig. 1K). Together, these findings suggest that lncRNAs commonly altered by *MAPT* mutations exhibit broad gene regulatory roles.

### Mutant tau-regulated lncRNAs are enriched in RNA-binding proteins that function in stress granule formation

LncRNAs can also act as scaffolds or decoys to promote or weaken the interaction between macromolecules [45]. The interaction of lncRNAs with RNA-binding proteins affects posttranslational modifications, stability, subcellular localization, and activity of interacting partners [46]. RNA-binding proteins typically consist of aggregation-promoting, low complexity domains, or prion-like domains, and are involved in the formation of stress granules [47–51]. Tau interacts with a number of RNA-binding proteins in vitro and in vivo that then facilitate stress granule formation, which may act as precursors to tau aggregates in FTLD-tau [52].

**Fig. 2:**
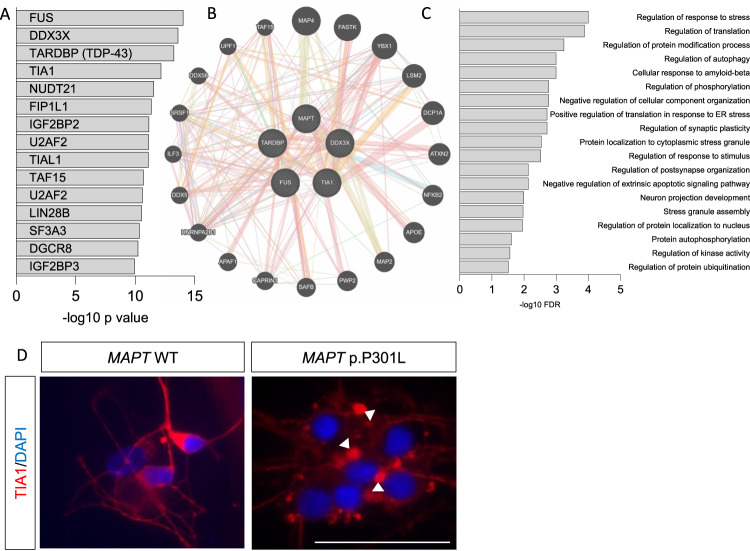
Common differentially expressed long non-coding RNAs (lncRNAs) interact with RNA-binding proteins and are involved in stress granule formation. **A** Common differentially expressed lncRNAs are predicted to interact with RNA-binding proteins. **B** GeneMANIA network of the most significant RNA-binding proteins *FUS*, *DDX3X*, *TARDBP*, *TIA1*, and *MAPT*. **C** GO terms obtained from the network using STRING. **D** TIA1-positive stress granules (red) are detectable in *MAPT* p.P301L neurons. White arrows indicate stress granules. Scale bar, 50 µm.

To further examine the molecular functions of the 15 common lncRNAs, we evaluated the potential interaction with RNA-binding proteins using the lncSEA algorithm [27]. The mutant tau-regulated lncRNAs were found to interact with 255 RNA-binding proteins, the top 15 are shown in Fig. 2A (see also Supplementary Table 10). Interestingly, FUS, DDX3X, TARDBP (encoding TDP-43 protein), and TIA1 were predicted to be the most significant interacting partners (Fig. 2A). *FUS* and *TARDBP* are FTLD genes, and DDX3X and TIA1 have been implicated in tauopathies [47, 50, 51, 53]. Gene network analysis of *FUS*, *DDX3X*, *TARDBP*, *TIA1*, and *MAPT* reveal that *TIA1*, *FUS*, *YBX1*, *ATXN2*, *APOE*, *MAP2*, *PWP2*, *APAF1*, *HNRNPA2B1*, and *ILF3* physically interact with *MAPT* (Fig. 2B). Interactions between *MAP4*, *DDX3X*, *FUS*, *TARDBP*, *HNRNPA2B1*, *ILF3*, and *MAPT* have been experimentally validated (Fig. 2B). *FUS*, *DDX3X*, *TARDBP*, *TIA1*, and *MAPT* function in pathways related to the regulation of response to stress (FDR = 1 × 10^−4^), regulation of translation (FDR = 1.3 × 10^−4^), regulation of autophagy (FDR = 1 × 10^−3^), and stress granule assembly (FDR = 1.06 × 10^−2^; Fig. 2C). Thus, the 15 common lncRNAs interact with RNA-binding proteins that mediate stress granule formation.

Given our observation that common differentially expressed lncRNAs are enriched in RNA-binding proteins that regulate stress granule formation, we evaluated stress granule formation in iPSC-derived neurons from *MAPT* p.P301L and isogenic controls using immunocytochemistry for stress granule marker, TIA1 (Fig. 2D). We observed that *MAPT* mutant neurons have a marked increase in TIA1-positive stress granules. This is consistent with prior reports in mouse models of tauopathy (P301L-Tg4510 and PS19) and human FTLD-tau patients [54, 55]. Thus, *MAPT* mutations lead to the accumulation of stress granules in human neurons.

### *SNHG8* is dysregulated in a mouse model of tauopathy and in human brain tissue

**Fig. 3:**
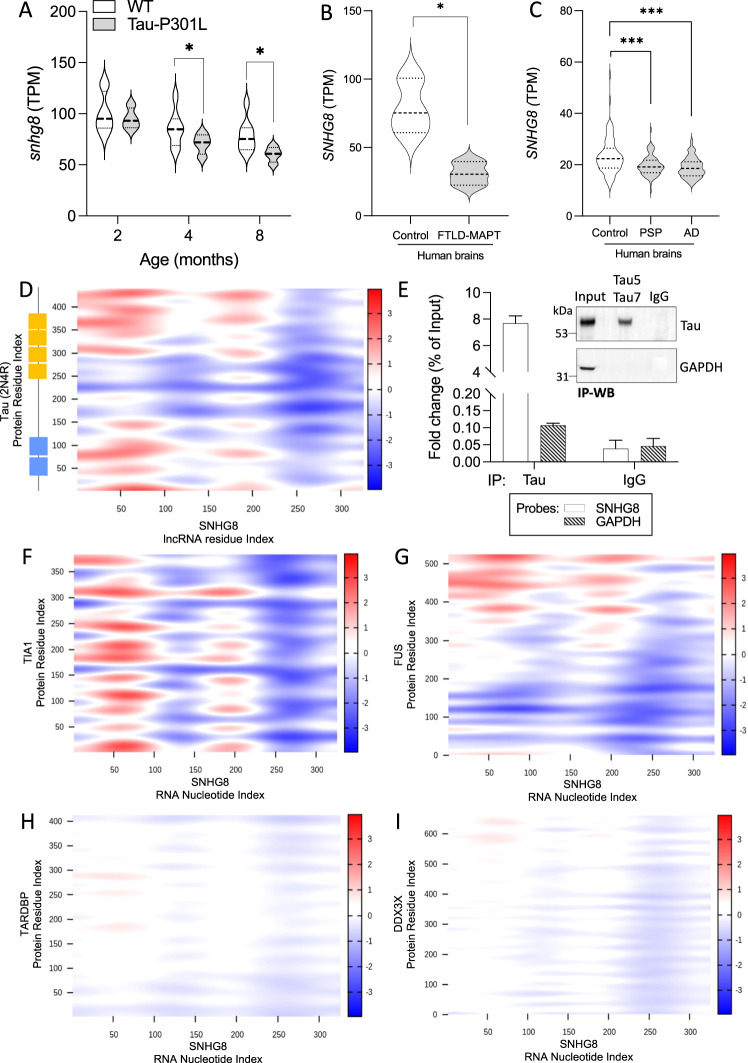
*SNHG8* is downregulated in mouse and human brains and interacts with tau, TIA1, FUS, TARDBP, and DDX3X. **A** Normalized read counts (TPM) of *Snhg8* in WT mice and Tau-P301L mice. **B** Normalized read counts of *SNHG8* in control and FTLD-tau (FTLD-MAPT, *MAPT* IVS10 + 16, and *MAPT* p.P301L carrier) brains. *p ≤ 0.05. **C** Normalized read counts of *SNHG8* in control, progressive supranuclear palsy (PSP), and Alzheimer’s disease (AD) brains. ***p ≤ 0.001. **D** CatRAPID interaction profile of 2N4R tau reveals multiple interaction domains with *SNHG8*. **E** RNA pull down to measure the interaction between tau and *SNHG8* using WT-Tau-(2N4R) transfected HEK293-T cells. Plot shows relative transcript expression of *SNHG8* and *GAPDH* after pull-down with tau antibodies (Tau5 and Tau7) or IgG, control. Data are representative of 3 independent experiments. Bar graph is represented as mean ± SEM. Student’s paired *t* test was used to determine significance. *p ≤ 0.05. **F**–**I** CatRAPID interaction profile of RNA-binding proteins with *SNHG8*, the colors in the heatmap indicate the interaction score (ranging from −3 to +3) of the individual amino acid and nucleotide pairs.

Despite many strengths of human stem cell models, there remains a need to validate key discoveries in a dish using in vivo models to prioritize those changes that are relevant to disease phenotypes. Thus, we sought to determine the extent to which the 15 commonly differentially expressed lncRNAs across *MAPT* mutations (Fig. 1H, I) were altered as tau accumulates in the Tau-P301L mouse model of tauopathy. Using the Mouse Dementia Network, we analyzed transcriptomic data generated from the cortex of WT and Tau-P301L mice collected at 2, 4, and 8 months [34, 35] (Fig. 3A). Among the 15 lncRNAs, only *Norad* (2900097C17Rik) and *Snhg8* were present in the dataset (Supplementary Table 11). The remaining 13 lncRNAs exhibit lower conservation among mammals and were not identified in the dataset. *Norad* expression was similar between WT and Tau-P301L at 2 and 4 months but was significantly elevated in Tau-P301L brains at 8 months, discordant with the findings in iPSC-neurons (Supplementary Fig. 2). At 2 months of age, *Snhg8* expression was similar between WT and Tau-P301L (Fig. 3A), suggesting that altered *Snhg8* expression was not developmentally encoded. At 4 and 8 months, *Snhg8* was significantly reduced in Tau-P301L mice (Fig. 3A). The reduced expression of *Snhg8* at 4 and 8 months of age coincides with periods of active accumulation of tau aggregation in Tau-P301L mice [35].

Leveraging isogenic iPSC lines to understand the contribution of a single allele to downstream phenotypes is a powerful system that, when applied here, has revealed lncRNAs shared across *MAPT* mutations that also change with tau accumulation in mouse models of tauopathy. However, a limitation of this approach is that iPSC-neurons are cultured in a dish and remain relatively immature. For example, iPSC-neurons predominantly express 0N3R tau [23, 56, 57], while the adult brain expresses 6 tau isoforms [23, 58]. Thus, we sought to determine whether *SNHG8* is altered in human brains of tauopathy patients. *SNHG8* was significantly reduced in FTLD-tau caused by *MAPT* IVS10 + 16 or p.P301L (Fig. 3B, Supplementary Table 12); a primary sporadic tauopathy, progressive supranuclear palsy (Fig. 3C, Supplementary Table 13); and a secondary tauopathy, Alzheimer’s disease (Fig. 3C, Supplementary Table 14). Expression of *SNHG8* was also validated by qPCR in iPSC-derived neurons expressing *MAPT* IVS10 + 16, p.P301L and p.R406W mutations and their isogenic controls (Supplementary Fig. 3). Together, we show *SNHG8* is altered in iPSC and mouse models of tauopathy and in tauopathy patient brains, supporting a role for *SNHG8* in pathologic processes.

### *SNHG8* interacts with tau and RNA-binding proteins

Given our findings of *SNHG8* dysregulation in iPSC-derived neurons from *MAPT* mutation carriers, mouse brains with tau aggregation, and human brains from tauopathy patients, we asked whether this effect occurs via direct interaction between tau and *SNHG8*. CatRAPID, a bioinformatic platform that predicts interactions between protein and RNA based on structure data, was employed [30]. Several domains of the longest tau isoform (2N4R) are predicted to interact with *SNHG8* (Fig. 3D). To functionally validate the predicted interaction between tau and *SNHG8* in vitro, HEK293-T cells were transiently transfected with plasmids containing untagged WT-Tau (2N4R). RNA-IP was performed using total tau antibodies (Tau5 and Tau7) to enrich for tau. qPCR of the RNA fraction bound to total tau revealed a ~6-fold enrichment of *SNHG8* in the tau fraction compared with the IgG, control fraction (*p* < 0.001; Fig. 3E). Thus, *SNHG8* interacts with tau protein.

Common differentially expressed lncRNAs were enriched in RNA-binding proteins TIA1, FUS, DDX3X, and TDP-43 (Fig. 2A); so, we asked whether *SNHG8* interacts with these RNA-binding proteins and where the interaction occurs using CatRAPID. *SNHG8* is predicted to bind to regions of TIA1, FUS, DDX3X, and TDP-43 (Fig. 3F–I). TIA1 showed the strongest interaction with *SNHG8* (interaction propensity: 65; Fig. 3F). FUS, DDX3X, and TDP-43 interact with *SNHG8* to a lesser extent but passed the threshold for positive interaction (interaction propensity: 45, 37, and 14, respectively; Fig. 3G–I).

### Mutant tau and stress drive stress granule formation via *SNHG8*

**Fig. 4:**
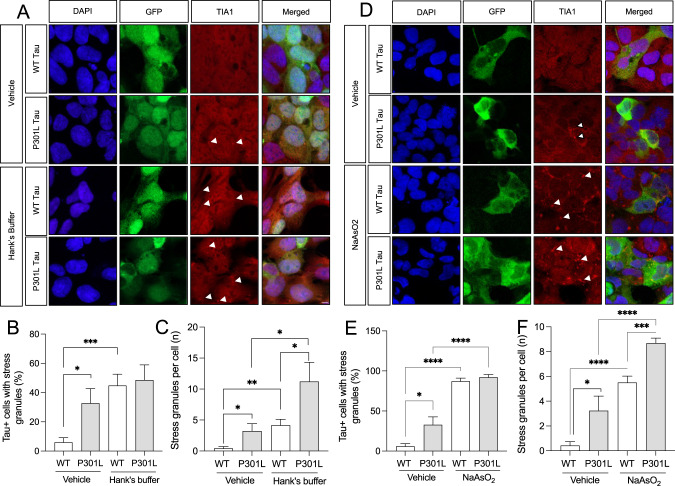
Mutant tau and stress enhance stress granule formation. **A** Immunocytochemistry of HEK293-T cells transiently transfected with WT-Tau-GFP or P301L-Tau-GFP and exposed to basal conditions (vehicle) and nutrient deprivation (Hank’s buffer). Tau (GFP, green), TIA1 (red). White arrow, stress granules. Scale bar, 5 um. **B** Bar graph representing the quantification of Tau-positive cells with TIA1-positive stress granules. **C** Bar graph of the number of stress granules in Tau-positive cells. White bars, WT-Tau-GFP expressing cells. Gray bars, P301L-Tau-GFP expressing cells. **D** Immunocytochemistry of HEK293-T cells transiently transfected with WT-Tau-GFP or P301L-Tau-GFP and exposed to basal conditions (vehicle) or oxidative stress (NaAsO_2_). tau (GFP, green), TIA1 (red). White arrow, stress granules. Scale bar, 5 um. **E** Bar graph representing the quantification of tau-positive cells with TIA1-positive stress granules. **F** Bar graph of the number of stress granules in tau-positive cells. The data represents at least 4 independent experiments. Bar graphs represent mean ± SEM. Statistical significance is determined with a Student’s *t* test. *p ≤ 0.05; **p ≤ 0.001, and ***p ≤ 0.0001.

TIA1 has been shown to interact with tau and to facilitate its incorporation into stress granules [54, 59]. To evaluate the impact of a representative *MAPT* mutation on stress granule formation in vitro, we compared HEK293-T cells in which plasmids containing WT-Tau-GFP or P301L-Tau-GFP were transiently overexpressed (Fig. 4). TIA1, a stress granule resident protein, was used as a marker of stress granule accumulation. TIA1 and tau co-localized in this model; however, not all TIA1-positive stress granules were positive for tau. Under basal conditions, the percentage of tau-positive cells with TIA1-positive stress granules and the number of stress granules per cell was significantly increased in the P301L-Tau-GFP expressing cells compared with WT-Tau-GFP (*p* < 0.05; Fig. 4A–C).

Next, we explored whether there was a differential genotypic impact on stress granule formation with stress induction via nutrient deprivation or oxidative stress. HEK293-T cells were transfected with WT-Tau-GFP or P301L-Tau-GFP and cultured in Hank’s buffer (nutrient deprivation) or 0.5 mM NaAsO_2_ (oxidative stress) for 1 h prior to immunocytochemistry. Stress induction by nutrient deprivation was sufficient to produce an increase in the percentage of TIA1-positive stress granules in WT-Tau-GFP and P301L-Tau-GFP expressing cells (Fig. 4A–C). Cells expressing P301L-Tau-GFP also produced significantly more TIA1-positive stress granules per cell under basal conditions than WT-Tau-GFP expressing cells (*p* < 0.05; Fig. 4A–C). To evaluate the impact of oxidative stress and *MAPT* genotype on stress granule formation, we treated WT-Tau-GFP and P301L-Tau-GFP expressing cells with 0.5 mM NaAsO_2_ for 1 h. Stress granule formation was analyzed by immunostaining for TIA1 and a second marker of stress granules, PABP, a poly-A binding protein that recruits RNA-binding proteins and non-RNA-binding proteins to the stress granule [60] (Fig. 4D; Supplementary Fig. 4A). Oxidative stress produced a significant increase in the percentage of cells with TIA1-positive and PABP-positive stress granules and the number of stress granules per cell in both WT-Tau-GFP and P301L-Tau-GFP expressing cells (Fig. 4D-E; Supplementary Fig. 4A–C). Additionally, in the presence of oxidative stress, cells expressing P301L-Tau-GFP produced significantly more TIA1-positive stress granules per cell compared with WT-Tau-GFP expressing cells (Fig. 4D–F; Supplementary Fig. 4A–C). Together, these findings suggest that mutant tau enhances stress granule formation under basal and stress conditions.

**Fig. 5:**
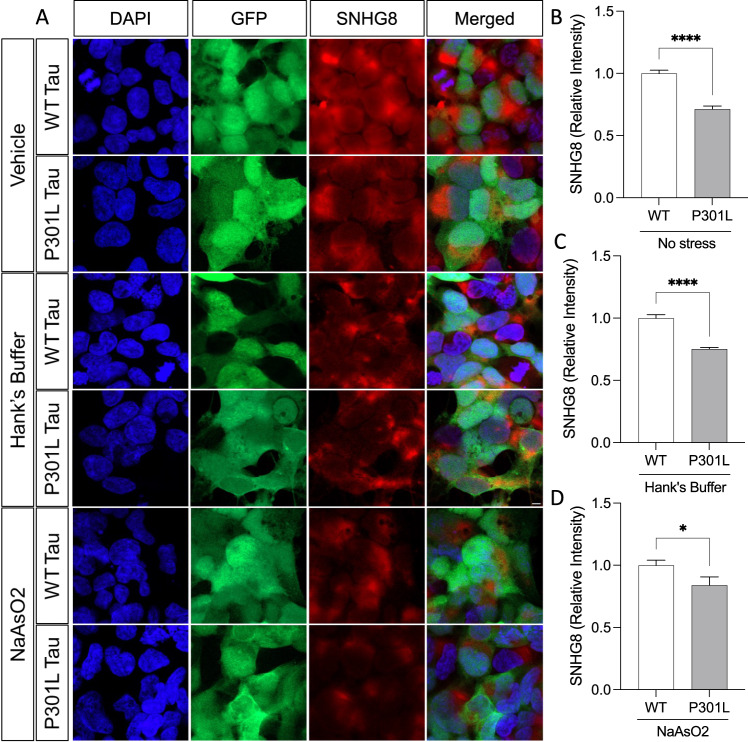
Mutant tau and stress lead to downregulation of *SNHG8*. **A** RNAscope for *SNHG8* in WT-Tau-GFP and P301L-Tau-GFP-expressing HEK293-T cells under basal conditions (vehicle) and stress (Hank’s buffer, nutrient deprivation or NaAsO_2_, oxidative stress). tau (GFP, green), *SNHG8* (red). Scale bar, 10 um. **B**–**D** Bar graph representing the quantification of mean intensity of *SNHG8* in tau-positive cells. Data is representative of at least 4 independent experiments. Bar graphs represent mean ± SEM. Statistical significance was determined using a Student’s *t* test. ***p ≤ 0.0001, **p < 0.001, *p < 0.05).

To examine endogenous *SNHG8* expression in the presence of mutant tau and upon stress induction, *SNHG8* levels were monitored using RNAscope. In the absence of stress, *SNHG8* was significantly reduced in cells expressing P301L-Tau-GFP compared to WT-Tau-GFP (*p* < 0.0001; Fig. 5A, B; control probes shown in Supplementary Fig. 5). Furthermore, expression of mutant tau (P301L-Tau-GFP) led to significantly reduced *SNHG8* upon mild (Hank’s buffer) and robust (NaAsO_2_) stress induction compared to WT-Tau-GFP expressing cells (*p* < 0.05; Fig. 5). Interestingly, we also observed that stress induction (Hank’s buffer or NaAsO_2_) was sufficient to reduce *SNHG8* in WT-Tau-GFP expressing cells (Supplementary Fig. 6). Thus, stress induction and mutant tau are sufficient to reduce *SNHG8* levels.

### *SNHG8* blocks stress granule formation

**Fig. 6:**
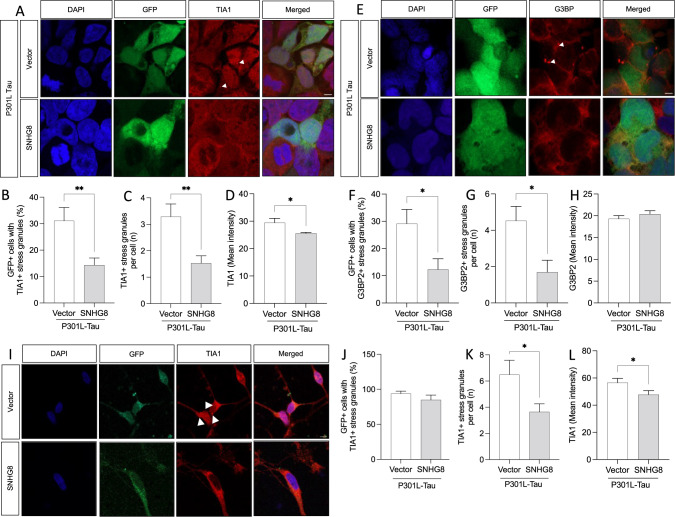
Overexpression of *SNHG8* inhibits stress granule formation in cells expressing mutant tau. Immunocytochemistry of HEK293-T cells co-overexpressing P301L-Tau with GFP (vector) or *SNHG8*-GFP under basal conditions. **A** GFP (green), TIA1 (red). White arrow, stress granules. Scale bar, 50 um. **B** Bar graph representing the quantification of GFP-positive cells with TIA1-positive stress granules. **C** Bar graph of the number of TIA1-positive stress granules in GFP-positive cells. **D** Bar graph representing mean intensity of TIA1. **E** GFP (green), G3BP2 (red). White arrow, stress granules. Scale bar, 50 um. **F** Bar graph representing the quantification of GFP-positive cells with G3BP2-positive stress granules. **G** Bar graph of the number of G3BP2-positive stress granules in GFP-positive cells. **H** Bar graph representing mean intensity of G3BP2. **I** *SNHG8* overexpression reduces stress granule formation in iPSC-derived neurons expressing *MAPT* p.P301L. GFP (vector) or *SNHG8*-GFP were nucleofected in neural progenitor cells and cells were subsequently differentiated into neurons for 42 days. Immunocytochemistry for GFP (green) and TIA1 (red). White arrow, stress granules. Scale bar, 50 um. **J** Bar graph representing the quantification of GFP-positive cells with TIA1-positive stress granules. **K** Bar graph of the number of TIA1-positive stress granules in GFP-positive cells. **L** Bar graph representing mean intensity of TIA1. Data are representative of 4 independent experiments. Bar graphs represent mean ± SEM. Student’s *t* test was performed to determine statistical significance. *p ≤ 0.05, ***p ≤ 0.0001.

Given our findings that *SNHG8* interacts with tau and stress granule proteins along with evidence that mutant tau and stress induction regulate *SNHG8* levels, we asked whether rescuing *SNHG8* expression could reduce stress granule assembly in P301L-Tau-expressing cells. HEK293-T cells were co-transfected with P301L-Tau and *SNHG8-*GFP or GFP vector control (vector). This resulted in a 5-fold increase in *SNHG8* transcript levels (Supplementary Fig. 7A, B). Overexpression of *SNHG8* was sufficient to reduce the percentage of cells with TIA1-positive stress granules and the number of TIA1-positive stress granules per cell (Fig. 6A–C). The impact of *SNHG8* on stress granule formation may be driven by repression of TIA1 expression, as TIA1 protein levels are significantly reduced in P301L-Tau-expressing cells (Fig. 6A, D). This effect occurs primarily at the protein level, as *TIA1* mRNA was similar between *SNHG8* and vector control (Supplementary Fig. 7C, D).

To determine whether *SNHG8* has a broad effect on stress granule formation, we evaluated G3BP2 and PABP levels, stress granule assembly factors. *SNHG8* overexpression led to reduced G3BP2 and PABP-positive stress granules in P301L-Tau expressing cells as compared to vector control (Fig. 6E–G; Supplementary Fig. 8A–C). Total PABP intensity was significantly reduced when *SNHG8* was overexpressed (Supplementary Fig. 8A, D). G3BP2 intensity, however, was similar between *SNHG8* expressing cells and vector control (Fig. 6E, H).

**Fig. 7:**
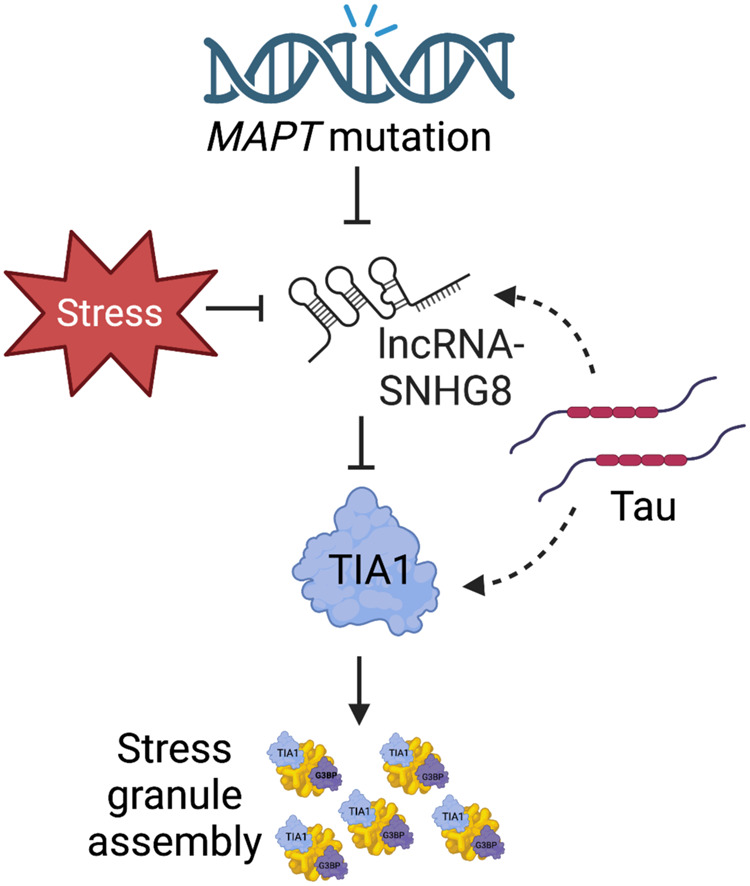
*SNHG8* is a regulator of stress granule formation in tauopathy. Schematic of major findings. *MAPT* mutations or stress led to repression of *SNHG8* expression. This repression limits *SNHG8* interaction with tau and enables tau to interact with TIA1 and form stress granules.

Finally, to demonstrate the degree to which these mechanisms are conserved in human neurons expressing mutant tau, we overexpressed *SNHG8* or vector control plasmids in iPSC-derived neurons from a *MAPT* p.P301L carrier and evaluated stress granule formation using TIA1. In human neurons, *SNHG8* was sufficient to reduce stress granule burden in mutant neurons (measured as number of stress granules/cell; Fig. 6I–K). *SNHG8* expression also led to significantly lower TIA1 protein levels in mutant neurons (Fig. 6I, L). Together, these studies suggest that *SNHG8* regulates stress granule formation by modifying TIA1 protein levels (Fig. 7).

## Discussion

The goal of this study was to investigate the regulatory potential of lncRNAs in tauopathies. Patient-derived iPSCs have served as a powerful tool for studying the molecular and cellular mechanisms of neurodegeneration. IPSC-derived neurons expressing *MAPT* IVS10 + 16, p.P301L, or p.R406W have been reported to recapitulate aspects of tau pathophysiology including elevated phosphorylated tau, disrupted endolysosomal function, impaired *MAPT* splicing, and altered synaptic function [5, 12, 15, 43]. Here, using iPSC-derived neurons expressing these same mutations, we identify 15 lncRNAs that are commonly differentially expressed across the *MAPT* IVS10 + 16, p.P301L, and p.R406W. These 15 lncRNAs function to regulate protein-coding gene expression in the human neurons and to interact with RNA-binding proteins involved in stress granule formation. Among these lncRNAs, *SNHG8* was significantly reduced in a mouse model of tauopathy and in the brains of patients with tauopathy, supporting a role for *SNHG8* in pathologic processes. We show that *SNHG8* interacts with tau, and overexpression of tau in vitro is sufficient to reduce *SNHG8* expression and induce TIA1-positive stress granule formation. Genetic manipulation of *SNHG8* leads to reduced stress granule formation suggesting that dysregulation of this non-coding RNA is a causal factor driving stress granule formation in tauopathies (Fig. 7).

LncRNAs can alter gene expression by cis or trans mechanisms. We found that the commonly differentially expressed lncRNAs were highly correlated with coding genes commonly differentially expressed in the presence of *MAPT* mutations [15]. Gene enrichment analyses identified pathways related to Neurotrophin trk receptor signaling, Notch signaling, BDNF signaling, lipoprotein lipase activity, and axonal guidance. The 275 protein-coding genes are enriched in pathways associated with neuronal processes, synaptic function, and endolysosomal function [15]. However, here, we find that differentially expressed lncRNAs impact pathways that are restricted to those related to neuronal processes. Synaptic dysfunction has been widely reported among *MAPT* mutation carriers and in FTLD-tau [8, 42]. Interestingly, the 15 lncRNAs are also associated with lipoprotein lipase activity, which point to a regulatory role in lipid metabolism. Cholesterol dyshomeostasis and the accumulation of lipid droplets have been reported in tauopathies [61]. Thus, dysregulation of lncRNAs may contribute to several disparate phenotypes observed in tauopathies.

Dysregulation of RNA-binding proteins has been linked to various neurological disorders, including ALS, FTLD, AD, Huntington’s disease (HD), and Creutzfeldt-Jakob disease (CJD) [62, 63]. The *TARDBP* gene encodes the TDP-43 protein, which is the primary component of pathological aggregates in most cases of ALS and 40% of cases of FTLD associated with progranulin haplo-insufficiency [64]. Similarly, mutations in *FUS*, *HNRNPA1/B2*, and other RNA-binding proteins have been associated with familial forms of motor neuron disorders [63]. Interaction of tau with RNA-binding proteins and ribosomes affects protein translation and RNA metabolism. In AD, the association of tau with RNA-binding proteins is increased, which may contribute to the dysregulation of RNA metabolism [65]. LncRNAs have also been shown to interact with a wide range of RNA-binding proteins, which can impact posttranslational modification, stability, subcellular localization, and activity of interacting partners [46, 66]. The 15 common lncRNAs identified in our study were predicted to interact with several RNA-binding proteins that have been previously implicated in neurodegeneration. RNA-binding proteins including FUS, TARDBP, and TIA1 have been shown to contribute to pathology in FTLD-tau and FTLD-TDP [47–51]. Thus, studying the impact of lncRNAs across neurodegenerative disease may reveal novel disease mechanisms.

Another hallmark of FTLD-tau is the presence of pathologic stress granules in neurons. Stress granules are cytoplasmic complexes that form in response to nutritional stress, DNA damage, and proteostatic dysfunction [38, 54, 67]. The liquid–liquid phase separation of stress granules is primarily driven by weak electrostatic, hydrophobic, and homo- and heterotypic protein–protein interactions between RNA-binding proteins that contain intrinsically disordered domains. Hence, identifying molecular drivers that affect the stability and assembly of stress granules is crucial to understanding basic molecular mechanisms of stress granule assembly and their role in the neuropathogenesis of FTLD-tau. The presence of stress granules has been reported in a mouse model of tauopathy and FTLD-tau patients [54]. Under physiological conditions, tau has been shown to selectively co-partition with the RNA-binding protein TIA1, which consists of intrinsically disordered domains or prion-like domains to form aggregates [47, 68]. However, molecular drivers involved in the regulation of stress granule assembly in tau neuropathology were unknown. Here, we provide evidence that *SNHG8* is a major regulator of TIA1-mediated stress granule formation. We find that when stress granules form, in the presence of mutant tau or upon stress induction, *SNHG8* levels are reduced. Additionally, rescuing *SNHG8* levels reduces stress granule formation and TIA1 protein levels. These findings are consistent with prior observations that downregulation of TIA1 inhibits stress granule formation [55] and tau accumulation [47, 54, 59]. Interestingly, *SNHG8* was among the RNAs enriched in the interacting transcriptome of WT-Tau and P301L-Tau aggregates isolated from HEK293 biosensor cells [52]. Interestingly, our findings that stress alone was sufficient to reduce *SNHG8* and promote stress granule formation provides a possible explanation for a sporadic tauopathy, PSP, where *SNHG8* is significantly reduced in PSP patient brains. Together, we provide novel insights into the mechanisms of stress granule assembly in the neuropathology of FTLD-tau via the *SNHG8*/TIA1 axis.

Small nucleolar RNA host genes (SNHGs) are a group of lncRNAs that contain introns and exons in their sequences and generate small nucleolar RNAs through alternative splicing. *SNHG8* is a newly identified type of small nucleolus host RNA belonging to the long intergenic non-coding RNA family, located on chromosome 4q26 [69, 70]. *SNHG8* is expressed by most cell types in the brain including neurons, oligodendrocytes, microglia, and astrocytes [71]. *SNHG8* has been largely studied in the context of tumorigenesis, where it has been shown to promote the proliferation and invasion of cancer cells in gastric cancer, breast cancer, and ovarian cancer [72–74]. The role of *SNHG8* in brain function and disease is not well investigated. Recently, *SNHG8* was reported to be involved in inflammation and microglial response by sponging miR-425-5p and SIRT1/NF-κB signaling [75] and in orthodontic tooth movement [76]. *SNHG8* has been associated with an inflammatory response: reducing *SNHG8*, which binds to HIF-1α, leads to free functional HIF-1α and activation of the downstream NF-κB pathway [76]. The consideration of therapeutic strategies involving *SNHG8* replacement will require an evaluation of the landscape of *SNHG8* effects.

This study focused on those lncRNAs that were commonly differentially expressed across *MAPT* mutation types and associated with tau pathological events in vivo. This approach allowed us to focus on those lncRNAs that are most relevant to disease processes. However, lncRNAs are not fully annotated in many of the publicly available datasets, limiting our validation and translational potential. Therefore, additional investigation of lncRNAs in neurodegeneration will be important. Additionally, the lncRNA discovery was made in iPSC-derived neurons, which lack aspects of late stage tauopathy such as tau aggregation and neurodegeneration. Thus, there remains additional work to understand the contribution of lncRNAs in early and late stages of tau pathophysiology. The impact of stress granules on cellular function remains to be fully resolved. The contribution of stress granules to the formation of protein aggregates observed in neurodegeneration is not well understood but may serve to seed prion-like assembly of beta-sheet rich protein [77]. Together, our study provides novel insights into the role of lncRNAs in pathological events leading to tauopathy. We show that lncRNA *SNHG8* is an interacting partner of tau, and *SNHG8* is a biological inhibitor of stress granule assembly via TIA1.

### Supplementary information

Supplemental Table 1

Supplemental Table 2

Supplemental Table 3

Supplemental Table 4

Supplemental Table 5

Supplemental Table 6

Supplemental Table 7

Supplemental Table 8

Supplemental Table 9

Supplemental Table 10

Supplemental Table 11

Supplemental Table 12

Supplemental Table 13

Supplemental Table 14

Supplemental Figure 1

Supplemental Figure 2

Supplemental Figure 3

Supplemental Figure 4

Supplemental Figure 5

Supplemental Figure 6

Supplemental Figure 7

Supplemental Figure 8

## References

[1] Bodea LG, Eckert A, Ittner LM, Piguet O, Götz J (2016). Tau physiology and pathomechanisms in frontotemporal lobar degeneration. J Neurochem.

[2] Pottier C, Ravenscroft TA, Sanchez-Contreras M, Rademakers R (2016). Genetics of FTLD: overview and what else we can expect from genetic studies. J Neurochem.

[3] Van Swieten J, Spillantini MG (2007). Hereditary frontotemporal dementia caused by Tau gene mutations. Brain Pathol.

[4] Zhu M, Zhang S, Tian X, Wu C. Mask mitigates MAPT- and FUS-induced degeneration by enhancing autophagy through lysosomal acidification. Autophagy. 2017;13:1924–38. 10.1080/15548627.2017.1362524. 10.1080/15548627.2017.1362524 PMC5788473 28806139

[5] Mahali S, Martinez R, King M, Verbeck A, Harari O, Benitez BA (2022). Defective proteostasis in induced pluripotent stem cell models of frontotemporal lobar degeneration. Transl Psychiatry.

[6] Caballero B, Wang Y, Diaz A, Tasset I, Juste YR, Stiller B (2018). Interplay of pathogenic forms of human tau with different autophagic pathways. Aging Cell.

[7] Frost B, Bardai FH, Feany MB (2016). Lamin dysfunction mediates neurodegeneration in tauopathies. Curr Biol.

[8] Tracy TE, Madero-Pérez J, Swaney DL, Chang TS, Moritz M, Konrad C (2022). Tau interactome maps synaptic and mitochondrial processes associated with neurodegeneration. Cell.

[9] Pradeepkiran JA, Hemachandra Reddy P (2020). Defective mitophagy in Alzheimer’s disease. Ageing Res Rev.

[10] Simone R, Javad F, Emmett W, Wilkins OG, Almeida FL, Barahona-Torres N (2021). MIR-NATs repress MAPT translation and aid proteostasis in neurodegeneration. Nature.

[11] Yan Y, Yan H, Teng Y, Wang Q, Yang P, Zhang L (2020). Long non-coding RNA 00507/miRNA-181c-5p/TTBK1/MAPT axis regulates tau hyperphosphorylation in Alzheimer’s disease. J Gene Med.

[12] Jiang S, Wen N, Li Z, Dube U, Del Aguila J, Budde J (2018). Integrative system biology analyses of CRISPR-edited iPSC-derived neurons and human brains reveal deficiencies of presynaptic signaling in FTLD and PSP. Transl Psychiatry.

[13] Bowles KR, Silva MC, Whitney K, Bertucci T, Berlind JE, Lai JD, et al. ELAVL4, splicing, and glutamatergic dysfunction precede neuron loss in MAPT mutation cerebral organoids. Cell. 2021;184:4547–63.e17. 10.1016/j.cell.2021.07.003. 10.1016/j.cell.2021.07.003 PMC8635409 34314701

[14] Hernandez I, Luna G, Rauch JN, Reis SA, Giroux M, Karch CM, et al. A farnesyltransferase inhibitor activates lysosomes and reduces tau pathology in mice with tauopathy. Sci Transl Med. 2019;11. 10.1126/scitranslmed.aat3005. 10.1126/scitranslmed.aat3005 PMC7961212 30918111

[15] Minaya MA, Mahali S, Iyer AK, Eteleeb AM, Martinez R, Huang G (2023). Conserved gene signatures shared among MAPT mutations reveal defects in calcium signaling. Front Mol Biosci.

[16] Oo JA, Brandes RP, Leisegang MS (2022). Long non-coding RNAs: novel regulators of cellular physiology and function. Pflug Arch Eur J Physiol.

[17] Zhang X, Wang W, Zhu W, Dong J, Cheng Y, Yin Z (2019). Mechanisms and functions of long non-coding RNAs at multiple regulatory levels. Int J Mol Sci.

[18] Khong A, Matheny T, Jain S, Mitchell SF, Wheeler JR, Parker R. The stress granule transcriptome reveals principles of mRNA accumulation in stress granules. Mol Cell. 2017;68:808–20.e5. 10.1016/j.molcel.2017.10.015. 10.1016/j.molcel.2017.10.015 PMC5728175 29129640

[19] Van Treeck B, Protter DSW, Matheny T, Khong A, Link CD, Parker R (2018). RNA self-assembly contributes to stress granule formation and defining the stress granule transcriptome. Proc Natl Acad Sci USA.

[20] Karch CM, Kao AW, Karydas A, Onanuga K, Martinez R, Argouarch A, et al. A comprehensive resource for induced pluripotent stem cells from patients with primary tauopathies. Stem Cell Rep. 2019;13:939–55. 10.1016/j.stemcr.2019.09.006. 10.1016/j.stemcr.2019.09.006 PMC6895712 31631020

[21] Takahashi K, Yamanaka S. Induction of pluripotent stem cells from mouse embryonic and adult fibroblast cultures by defined factors. Cell. 2006;126:663–76. 10.1016/j.cell.2006.07.024. 10.1016/j.cell.2006.07.024 16904174

[22] Ban H, Nishishita N, Fusaki N, Tabata T, Saeki K, Shikamura M (2011). Efficient generation of transgene-free human induced pluripotent stem cells (iPSCs) by temperature-sensitive Sendai virus vectors. Proc Natl Acad Sci USA.

[23] Sato C, Barthélemy NR, Mawuenyega KG, Patterson BW, Gordon BA, Jockel-Balsarotti J, et al. Tau kinetics in neurons and the human central nervous system. Neuron. 2018;97:1284–98.e7. 10.1016/j.neuron.2018.02.015. 10.1016/j.neuron.2018.02.015 PMC6137722 29566794

[24] Patro R, Duggal G, Love MI, Irizarry RA, Kingsford C (2017). Salmon provides fast and bias-aware quantification of transcript expression. Nat Methods.

[25] Love MI, Huber W, Anders S (2014). Moderated estimation of fold change and dispersion for RNA-seq data with DESeq2. Genome Biol.

[26] Wilkinson L. ggplot2: elegant graphics for data analysis by WICKHAM, H. Biometrics. 2011;67:678–9. 10.1111/j.1541-0420.2011.01616.x.

[27] Chen J, Zhang J, Gao Y, Li Y, Feng C, Song C (2021). LncSEA: a platform for long non-coding RNA related sets and enrichment analysis. Nucleic Acids Res.

[28] Montojo J, Zuberi K, Rodriguez H, Kazi F, Wright G, Donaldson SL (2010). GeneMANIA cytoscape plugin: fast gene function predictions on the desktop. Bioinformatics.

[29] Armaos A, Colantoni A, Proietti G, Rupert J, Tartaglia GG (2021). CatRAPID omics v2.0: Going deeper and wider in the prediction of protein-RNA interactions. Nucleic Acids Res.

[30] Bellucci M, Agostini F, Masin M, Tartaglia GG (2011). Predicting protein associations with long noncoding RNAs. Nat Methods.

[31] Hoover BR, Reed MN, Su J, Penrod RD, Kotilinek LA, Grant MK, et al. Tau mislocalization to dendritic spines mediates synaptic dysfunction independently of neurodegeneration. Neuron. 2010;68:1067–81. 10.1016/j.neuron.2010.11.030. 10.1016/j.neuron.2010.11.030 PMC3026458 21172610

[32] Karch CM, Jeng AT, Goate AM (2012). Extracellular tau levels are influenced by variability in tau that is associated with tauopathies. J Biol Chem.

[33] Bierhoff H (2018). Analysis of lncRNA-protein interactions by RNA-protein pull-down assays and RNA immunoprecipitation (RIP). Methods Mol Biol..

[34] Ramsden M, Kotilinek L, Forster C, Paulson J, McGowan E, SantaCruz K, et al. Age-dependent neurofibrillary tangle formation, neuron loss, and memory impairment in a mouse model of human tauopathy (P301L). J Neurosci. 2005;25:10637–47. 10.1523/JNEUROSCI.3279-05.2005. 10.1523/JNEUROSCI.3279-05.2005 PMC6725849 16291936

[35] Matarin M, Salih DA, Yasvoina M, Cummings DM, Guelfi S, Liu W (2015). A Genome-wide gene-expression analysis and database in transgenic mice during development of amyloid or tau pathology. Cell Rep.

[36] Allen M, Carrasquillo MM, Funk C, Heavner BD, Zou F, Younkin CS, et al. Human whole genome genotype and transcriptome data for Alzheimer’s and other neurodegenerative diseases. Sci Data. 2016;3:160089. 10.1038/sdata.2016.89. 10.1038/sdata.2016.89 PMC5058336 27727239

[37] Gilks N, Kedersha N, Ayodele M, Shen L, Stoecklin G, Dember LM (2004). Stress granule assembly is mediated by prion-like aggregation of TIA-1. Mol Biol Cell.

[38] Kedersha N, Anderson P (2007). Mammalian stress granules and processing bodies. Methods Enzymol.

[39] Hanson KK, Mair GR (2014). Stress granules and plasmodium liver stage infection. Biol Open.

[40] Zhang K, Daigle JG, Cunningham KM, Coyne AN, Ruan K, Grima JC, et al. Stress granule assembly disrupts nucleocytoplasmic transport. Cell. 2018;173:958–971.e17. 10.1016/j.cell.2018.03.025. 10.1016/j.cell.2018.03.025 PMC6083872 29628143

[41] Brunello CA, Yan X, Huttunen HJ (2016). Internalized Tau sensitizes cells to stress by promoting formation and stability of stress granules. Sci Rep.

[42] Nakamura M, Shiozawa S, Tsuboi D, Amano M, Watanabe H, Maeda S, et al. Pathological progression induced by the frontotemporal dementia-associated R406W tau mutation in patient-derived iPSCs. Stem Cell Rep. 2019;13:684–99. 10.1016/j.stemcr.2019.08.011. 10.1016/j.stemcr.2019.08.011 PMC6829766 31543469

[43] Capano LS, Sato C, Ficulle E, Yu A, Horie K, Barthelemy NR, et al. Recapitulation of endogenous 4R tau expression and formation of insoluble tau in directly reprogrammed human neurons. SSRN Electron J. 2021. 10.2139/ssrn.3899434. 10.1016/j.stem.2022.04.018 PMC9176216 35659876

[44] Statello L, Guo CJ, Chen LL, Huarte M (2021). Gene regulation by long non-coding RNAs and its biological functions. Nat Rev Mol Cell Biol.

[45] Wang KC, Chang HY (2011). Molecular mechanisms of long noncoding RNAs. Mol Cell.

[46] Yang Y, Wen L, Zhu H (2015). Unveiling the hidden function of long non-coding RNA by identifying its major partner-protein. Cell Biosci.

[47] Ash PEA, Lei S, Shattuck J, Boudeau S, Carlomagno Y, Medalla M, et al. TIA1 potentiates tau phase separation and promotes generation of toxic oligomeric tau. Proc Natl Acad Sci USA. 2021;118. 10.1073/pnas.2014188118. 10.1073/pnas.2014188118 PMC7936275 33619090

[48] Gerstberger S, Hafner M, Ascano M, Tuschl T (2014). Evolutionary conservation and expression of human RNA-Binding proteins and their role in human genetic disease. Adv Exp Med Biol.

[49] Latimer CS, Keene CD, Kraemer BC, Liachko NF. TDP-43 promotes pathological tau phosphorylation and selective neurotoxicity in C. elegans. Alzheimers Dement. 2021;17. 10.1002/alz.058137.

[50] Montalbano M, McAllen S, Cascio FL, Sengupta U, Garcia S, Bhatt N, et al. TDP-43 and tau oligomers in Alzheimer’s disease, amyotrophic lateral sclerosis, and frontotemporal dementia. Neurobiol Dis. 2020;146:105130. 10.1016/j.nbd.2020.105130. 10.1016/j.nbd.2020.105130 PMC7703712 33065281

[51] Urwin H, Josephs KA, Rohrer JD, MacKenzie IR, Neumann M, Authier A (2010). FUS pathology defines the majority of tau-and TDP-43-negative frontotemporal lobar degeneration. Acta Neuropathol.

[52] Lester E, Ooi FK, Bakkar N, Ayers J, Woerman AL, Wheeler J, et al. Tau aggregates are RNA-protein assemblies that mislocalize multiple nuclear speckle components. Neuron. 2021;109:1675–91.e9. 10.1016/j.neuron.2021.03.026. 10.1016/j.neuron.2021.03.026 PMC8141031 33848474

[53] Lennox AL, Hoye ML, Jiang R, Johnson-Kerner BL, Suit LA, Venkataramanan S, et al. Pathogenic DDX3X mutations impair RNA metabolism and neurogenesis during fetal cortical development. Neuron. 2020;106:404–20.e8. 10.1016/j.neuron.2020.01.042. 10.1016/j.neuron.2020.01.042 PMC7331285 32135084

[54] Vanderweyde T, Apicco DJ, Youmans-Kidder K, Ash PEA, Cook C, Lummertz da Rocha E, et al.Interaction of tau with the RNA-binding protein TIA1 regulates tau pathophysiology and toxicity. Cell Rep. 2016;15:1455–66. 10.1016/j.celrep.2016.04.045. 10.1016/j.celrep.2016.04.045 PMC5325702 27160897

[55] Maziuk BF, Apicco DJ, Cruz AL, Jiang L, Ash PEA, da Rocha EL (2018). RNA binding proteins co-localize with small tau inclusions in tauopathy. Acta Neuropathol Commun.

[56] Patani R, Lewis PA, Trabzuni D, Puddifoot CA, Wyllie DJA, Walker R, et al. Investigating the utility of human embryonic stem cell-derived neurons to model ageing and neurodegenerative disease using whole-genome gene expression and splicing analysis. J Neurochem. 2012;122:738–51. 10.1111/j.1471-4159.2012.07825.x. 10.1111/j.1471-4159.2012.07825.x PMC3504076 22681703

[57] Sposito T, Preza E, Mahoney CJ, Setó-Salvia N, Ryan NS, Morris HR (2015). Developmental regulation of tau splicing is disrupted in stem cell-derived neurons from frontotemporal dementia patients with the 10 + 16 splice-site mutation in MAPT. Hum Mol Genet.

[58] Hefti MM, Farrell K, Kim SH, Bowles KR, Fowkes ME, Raj T (2018). High-resolution temporal and regional mapping of MAPT expression and splicing in human brain development. PLoS One.

[59] Piatnitskaia S, Takahashi M, Kitaura H, Katsuragi Y, Kakihana T, Zhang L (2019). USP10 is a critical factor for Tau-positive stress granule formation in neuronal cells. Sci Rep.

[60] Marcelo A, Koppenol R, de Almeida LP, Matos CA, Nóbrega C (2021). Stress granules, RNA-binding proteins and polyglutamine diseases: too much aggregation?. Cell Death Dis.

[61] Glasauer SMK, Goderie SK, Rauch JN, Guzman E, Audouard M, Bertucci T (2022). Human tau mutations in cerebral organoids induce a progressive dyshomeostasis of cholesterol. Stem Cell Rep.

[62] Gunawardana CG, Mehrabian M, Wang X, Mueller I, Lubambo IB, Jonkman JEN (2015). The human tau interactome: binding to the ribonucleoproteome, and impaired binding of the proline-to-leucine mutant at position 301 (P301L) to chaperones and the proteasome. Mol Cell Proteom.

[63] Maziuk B, Ballance HI, Wolozin B (2017). Dysregulation of RNA binding protein aggregation in neurodegenerative disorders. Front Mol Neurosci.

[64] Neumann M, Sampathu DM, Kwong LK, Truax AC, Micsenyi MC, Chou TT (2006). Ubiquitinated TDP-43 in frontotemporal lobar degeneration and amyotrophic lateral sclerosis. Science.

[65] Meier S, Bell M, Lyons DN, Rodriguez-Rivera J, Ingram A, Fontaine SN, et al. Pathological tau promotes neuronal damage by impairing ribosomal function and decreasing protein synthesis. J Neurosci. 2016;36:1001–7. 10.1523/JNEUROSCI.3029-15.2016. 10.1523/JNEUROSCI.3029-15.2016 PMC4719006 26791227

[66] McMillan PJ, Benbow SJ, Uhrich R, Saxton A, Baum M, Strovas T (2023). Tau-RNA complexes inhibit microtubule polymerization and drive disease-relevant conformation change. Brain.

[67] Mahboubi H, Stochaj U (2017). Cytoplasmic stress granules: dynamic modulators of cell signaling and disease. Biochim. Biophys. Acta Mol Basis Dis.

[68] Wolozin B, Ivanov P (2019). Stress granules and neurodegeneration. Nat Rev Neurosci.

[69] Yuan X, Yan Y, Xue M (2021). Small nucleolar RNA host gene 8: a rising star in the targets for cancer therapy. Biomed Pharmacother.

[70] Williams GT, Farzaneh F (2012). Are snoRNAs and snoRNA host genes new players in cancer. Nat Rev Cancer.

[71] Zhang Y, Chen K, Sloan SA, Bennett ML, Scholze AR, O’Keeffe S, et al. An RNA-sequencing transcriptome and splicing database of glia, neurons, and vascular cells of the cerebral cortex. J Neurosci. 2014;34:11929–47. 10.1523/JNEUROSCI.1860-14. 10.1523/JNEUROSCI.1860-14.2014 PMC4152602 25186741

[72] Zou C, Liao J, Hu D, Su Y, Lin H, Lin K, et al. SNHG8 promotes the progression of Epstein–Barr virus-associated gastric cancer via sponging miR-512-5p and targeting TRIM28. Front Oncol. 2021;11:734694. 10.3389/fonc.2021.734694. 10.3389/fonc.2021.734694 PMC8554152 34722282

[73] Yu B, Wang B, Wu Z, Wu C, Ling J, Gao X, et al. LncRNA SNHG8 promotes proliferation and inhibits apoptosis of diffuse large B-cell lymphoma via sponging miR-335-5p. Front Oncol. 2021;11:650287. 10.3389/fonc.2021.650287. 10.3389/fonc.2021.650287 PMC8017314 33816305

[74] Miao W, Lu T, Liu X, Yin W, Zhang H (2020). LncRNA SNHG8 induces ovarian carcinoma cells cellular process and stemness through Wnt/β-catenin pathway. Cancer Biomark.

[75] Tian J, Liu Y, Wang Z, Zhang S, Yang Y, Zhu Y (2021). LncRNA Snhg8 attenuates microglial inflammation response and blood–brain barrier damage in ischemic stroke through regulating miR-425-5p mediated SIRT1/NF-κB signaling. J Biochem Mol Toxicol.

[76] Wang C, Yang Q, Han Y, Liu H, Wang Y, Huang Y (2022). A reduced level of the long non-coding RNA SNHG8 activates the NF-kappaB pathway by releasing functional HIF-1alpha in a hypoxic inflammatory microenvironment. Stem Cell Res Ther.

[77] Protter DSW, Parker R (2016). Principles and properties of stress granules. Trends Cell Biol.

