# Supplementary material for: Long non-coding RNA SNHG8 drives stress granule formation in tauopathies
Source: Mol Psychiatry. 2023 Sep 21;28(11):4889–901. doi: 10.1038/s41380-023-02237-2 (PMC10914599; doi:10.1038/s41380-023-02237-2)
Supplement: Supplementary file 15 — Supplemental Figure 1 [file 41380_2023_2237_MOESM15_ESM.pdf]

# Supplemental Figure 1

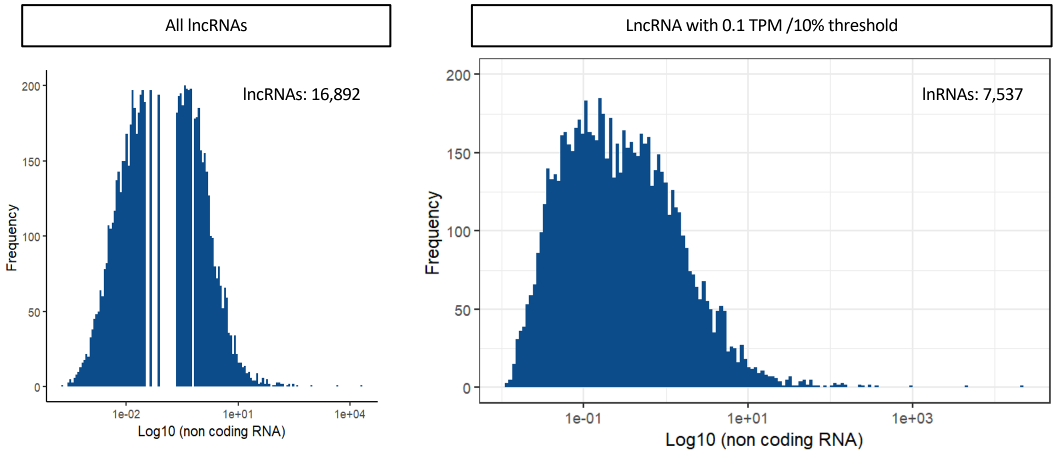


| Filter | Genes | Percentage |
|---|---|---|
| Total RNAs | 60,754 | 100% |
| Total number of non-coding RNAs | 16,892 | 26.8% |
| ≥0.1 TPM ≥10% threshold | 7,537 | 12.4% |

**Supplemental Figure 1: lncRNA distribution in sequenced neurons.** Left panel, distribution of lncRNA expression (including all lncRNAs). Right panel, after applying thresholds of 0.1 TPM expression in 10% of samples, histogram captures normal distribution. Table summarizing the numbers of genes at each stage. *TPM: transcripts per kilobase million. Normalized for gene length and for sequence depth.
