# Supplementary material for: Long non-coding RNA SNHG8 drives stress granule formation in tauopathies
Source: Mol Psychiatry. 2023 Sep 21;28(11):4889–901. doi: 10.1038/s41380-023-02237-2 (PMC10914599; doi:10.1038/s41380-023-02237-2)
Supplement: Supplementary file 16 — Supplemental Figure 2 [file 41380_2023_2237_MOESM16_ESM.pdf]

Supplemental Figure 2

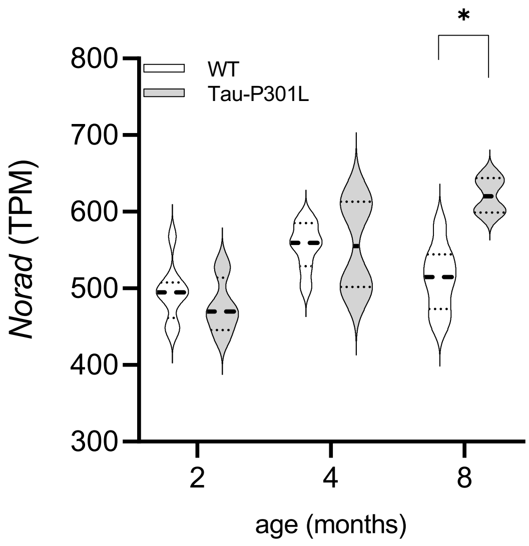

Supplemental Figure 2: *Norad* is differentially expressed in *MAPT* p.P301L mice. Normalized read counts (TPM) of *Norad* in WT mice and Tau-P301L mice. *, $p<0.05$.
