# Supplementary material for: Long non-coding RNA SNHG8 drives stress granule formation in tauopathies
Source: Mol Psychiatry. 2023 Sep 21;28(11):4889–901. doi: 10.1038/s41380-023-02237-2 (PMC10914599; doi:10.1038/s41380-023-02237-2)
Supplement: Supplementary file 17 — Supplemental Figure 3 [file 41380_2023_2237_MOESM17_ESM.pdf]

Supplemental Figure 3

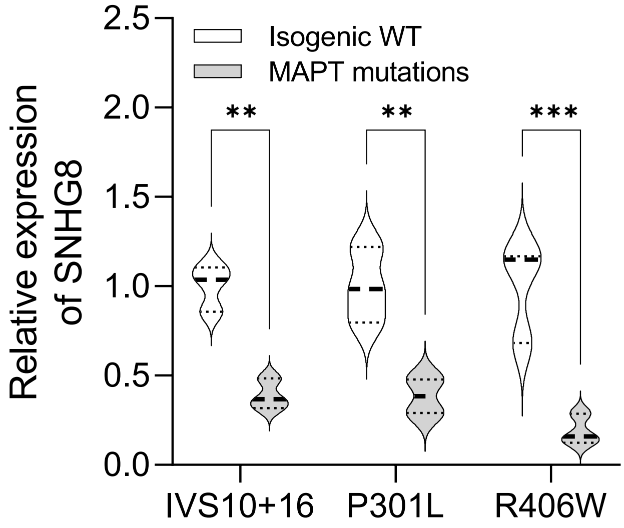

Supplementary Figure 3: qPCR validation of lncRNA SNHG8. qPCR analysis was performed using specific primers for SNHG8 (and GAPDH housekeeping gene) on cDNA from CRISPR corrected and *MAPT* mutant carrier neurons as indicated in the violin plots (n=3)(**p<0.01, ***p<0.001).
