# Supplementary material for: Long non-coding RNA SNHG8 drives stress granule formation in tauopathies
Source: Mol Psychiatry. 2023 Sep 21;28(11):4889–901. doi: 10.1038/s41380-023-02237-2 (PMC10914599; doi:10.1038/s41380-023-02237-2)
Supplement: Supplementary file 18 — Supplemental Figure 4 [file 41380_2023_2237_MOESM18_ESM.pdf]

Supplemental Figure 4

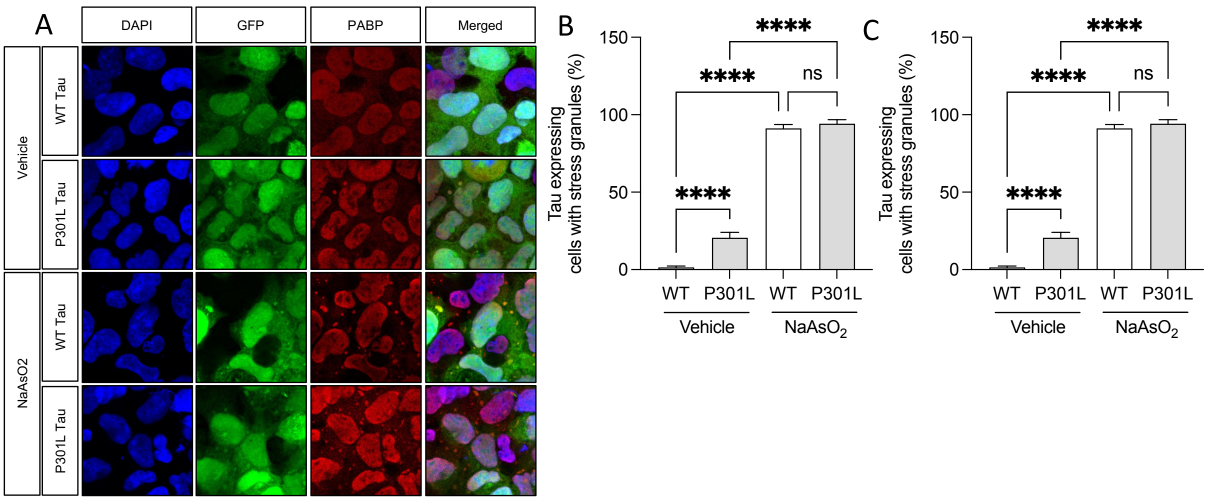

Supplemental Figure 4: P301L-Tau increases PABP-positive stress granules. A. Immunostaining of HEK293-T cells after transfection with WT-Tau-GFP or P301L-Tau-GFP and exposure to oxidative stress (sodium arsenite, $NaAsO_2$) or basal conditions (vehicle). B-C. Bar graphs showing quantification of Tau-expressing cells with stress granules as a percentage (B) and the number of stress granules per cell (C). Scale bar, 5µm * p<0.05, ****p<0.0001.
