# Supplementary material for: Long non-coding RNA SNHG8 drives stress granule formation in tauopathies
Source: Mol Psychiatry. 2023 Sep 21;28(11):4889–901. doi: 10.1038/s41380-023-02237-2 (PMC10914599; doi:10.1038/s41380-023-02237-2)
Supplement: Supplementary file 19 — Supplemental Figure 5 [file 41380_2023_2237_MOESM19_ESM.pdf]

Supplemental Figure 5

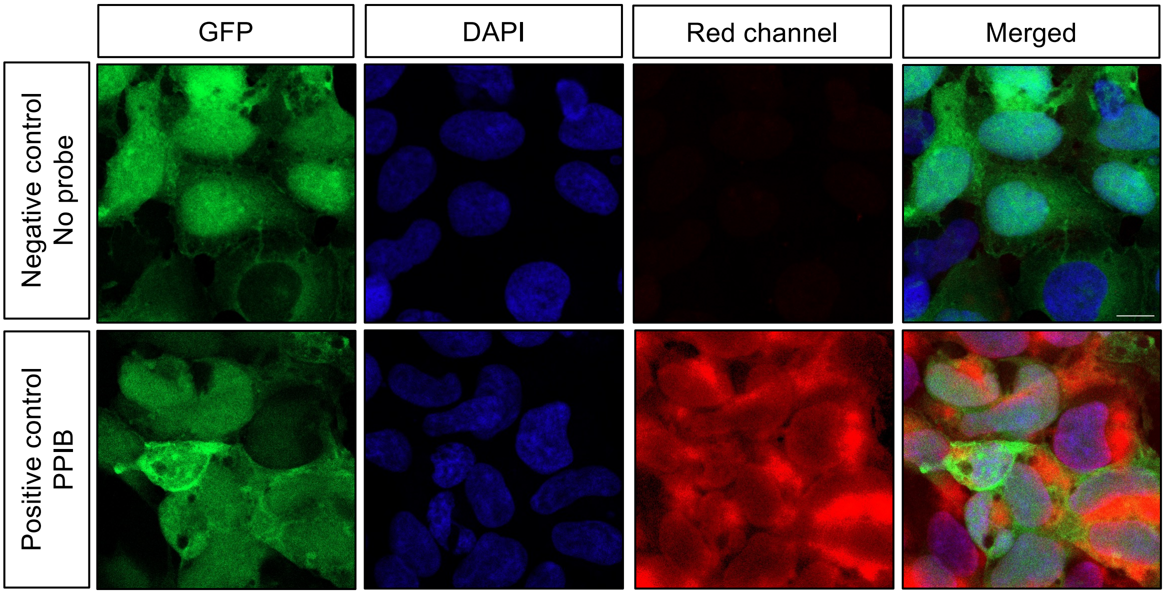

Supplemental Figure 5: RNAscope in HEK293-T cells. RNAscope using a negative control (no probe) and positive control (PPIB RNA probe). Scale bar, 10µm.
