# Supplementary material for: Long non-coding RNA SNHG8 drives stress granule formation in tauopathies
Source: Mol Psychiatry. 2023 Sep 21;28(11):4889–901. doi: 10.1038/s41380-023-02237-2 (PMC10914599; doi:10.1038/s41380-023-02237-2)
Supplement: Supplementary file 20 — Supplemental Figure 6 [file 41380_2023_2237_MOESM20_ESM.pdf]

Supplemental Figure 6

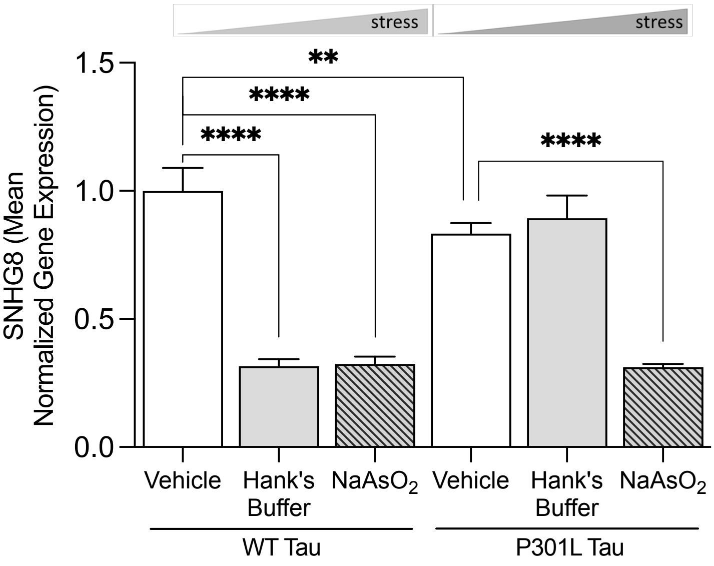

Supplemental Figure 6: Mutant tau and stress lead to downregulation of *SNHG8*. qPCR for *SNHG8* in WT-Tau-GFP and P301L-Tau-GFP-expressing HEK293-T cells under basal conditions (vehicle) and stress (Hank's buffer, nutrient deprivation or NaAsO2, oxidative stress). Bar graph showing relative expression of *SNHG8* measured by qPCR experiments. Data is representative of at least 4 independent experiments. Bar graphs represent mean ± SEM. Statistical significance was determined using a Student's t-test. p***≤0.0001, **p<0.001, *<0.05).
