# Supplementary material for: Long non-coding RNA SNHG8 drives stress granule formation in tauopathies
Source: Mol Psychiatry. 2023 Sep 21;28(11):4889–901. doi: 10.1038/s41380-023-02237-2 (PMC10914599; doi:10.1038/s41380-023-02237-2)
Supplement: Supplementary file 21 — Supplemental Figure 7 [file 41380_2023_2237_MOESM21_ESM.pdf]

Supplemental Figure 7

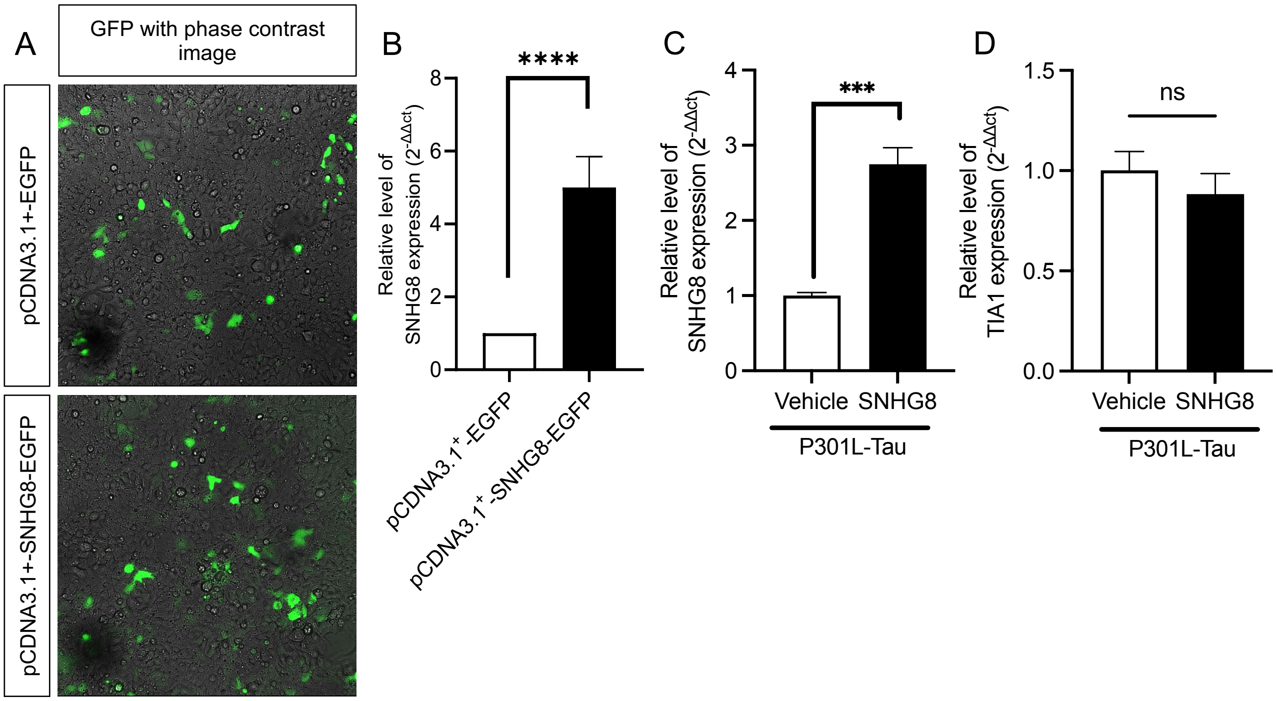

Supplemental Figure 7: Ectopic expression of *lncRNA-SNHG8* in HEK293T cells. A. Representative fluorescence images of HEK293T cells transfected with pCDNA3.1+-EGFP and pCDNA3.1+-SNHG8-EGFP plasmid constructs and images were taken 48hrs post transfection. The fluorescence images were overlayed with specific phase contrast image. B. Relative expression of *SNHG8* in HEK293T cells transfected with pCDNA3.1+-EGFP or pCDNA3.1+-SNHG8-EGFP plasmids. C-D. HEK293-T cells co-overexpressing P301L-Tau with GFP (vector) or SNHG8-GFP under basal conditions. Relative expression of *SNHG8* and *TIA1* measured by qPCR experiments. Expression normalized to *GAPDH*. n=4. Data are represented as mean ± SEM. **** $p < 0.0001$; unpaired Student’s t-test.
