# Supplementary material for: Long non-coding RNA SNHG8 drives stress granule formation in tauopathies
Source: Mol Psychiatry. 2023 Sep 21;28(11):4889–901. doi: 10.1038/s41380-023-02237-2 (PMC10914599; doi:10.1038/s41380-023-02237-2)
Supplement: Supplementary file 22 — Supplemental Figure 8 [file 41380_2023_2237_MOESM22_ESM.pdf]

Supplemental Figure 8

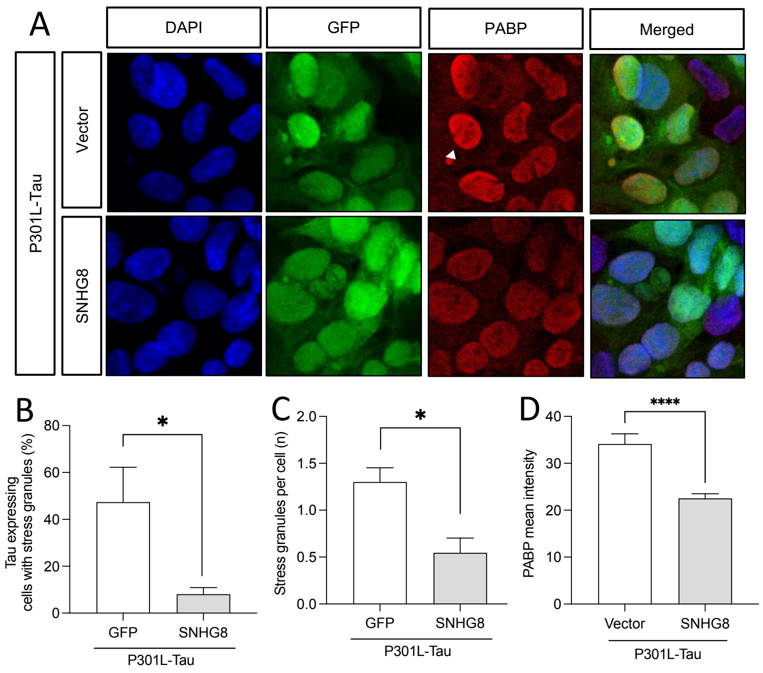

Supplemental Figure 8: *SNHG8* reduces PABP-positive stress granules. HEK293-T cells were transfected with WT-Tau-GFP or P301L-Tau-GFP under basal conditions. A. Immunostaining for PABP protein. PABP (red), GFP (green), and DAPI (blue). B-C. Bar graphs showing quantification of cells with stress granules as a percentage (B) and the number of stress granules per cell (C). D. PABP intensity. Scale, 5µm, *p<0.05.
